# Development of a Supported Self-management Intervention for People With Severe Mental Illness and Type 2 Diabetes: Theory and Evidence-Based Co-design Approach

**DOI:** 10.2196/43597

**Published:** 2023-05-12

**Authors:** Claire Carswell, Peter A Coventry, Jennifer V E Brown, Sarah L Alderson, Keith Double, Simon Gilbody, Richard I G Holt, Rowena Jacobs, Jennie Lister, David Osborn, David Shiers, Najma Siddiqi, Johanna Taylor, Ian Kellar, DIAMONDS Research Group

**Affiliations:** 1 Department of Health Sciences University of York York United Kingdom; 2 York Environmental Sustainability Institute University of York York United Kingdom; 3 Leverhulme Centre for Anthropocene Biodiversity University of York York United Kingdom; 4 Leeds Institute of Health Sciences University of Leeds Leeds United Kingdom; 5 Bradford District Care NHS Foundation Trust Bradford United Kingdom; 6 Centre for Health and Population Sciences Hull York Medical School York United Kingdom; 7 Human Development and Health Faculty of Medicine University of Southampton Southampton United Kingdom; 8 National Institute for Health Research Biomedical Research Centre University Hospital Southampton NHS Foundation Trust Southampton United Kingdom; 9 Centre for Health Economics University of York York United Kingdom; 10 Division of Psychiatry University College London London United Kingdom; 11 Psychosis Research Unit Greater Manchester Mental Health NHS Trust Manchester United Kingdom; 12 Division of Psychology and Mental Health University of Manchester Manchester United Kingdom; 13 School of Medicine Keele University Staffordshire United Kingdom; 14 Department of Psychology University of Sheffield Sheffield United Kingdom

**Keywords:** severe mental illness, diabetes, intervention development, co-design, mental health, comorbidity, mobile phone

## Abstract

**Background:**

Type 2 diabetes is 2 to 3 times more common among people with severe mental illness (SMI). Self-management is crucial, with additional challenges faced by people with SMI. Therefore, it is essential that any diabetes self-management program for people with SMI addresses the unique needs of people living with both conditions and the inequalities they experience within health care services.

**Objective:**

We combined theory, empirical evidence, and co-design approaches to develop a type 2 diabetes self-management intervention for people with SMI.

**Methods:**

The development process encompassed 4 steps: step 1 involved prioritizing the mechanisms of action (MoAs) and behavior change techniques (BCTs) for the intervention. Using findings from primary qualitative research and systematic reviews, we selected candidate MoAs to target in the intervention and candidate BCTs to use. Expert stakeholders then ranked these MoAs and BCTs using a 2-phase survey. The average scores were used to generate a prioritized list of MoAs and BCTs. During step 2, we presented the survey results to an expert consensus workshop to seek expert agreement with the definitive list of MoAs and BCTs for the intervention and identify potential modes of delivery. Step 3 involved the development of trigger films using the evidence from steps 1 and 2. We used animations to present the experiences of people with SMI managing diabetes. These films were used in step 4, where we used a stakeholder co-design approach. This involved a series of structured workshops, where the co-design activities were informed by theory and evidence.

**Results:**

Upon the completion of the 4-step process, we developed the DIAMONDS (diabetes and mental illness, improving outcomes and self-management) intervention. It is a tailored self-management intervention based on the synthesis of the outputs from the co-design process. The intervention incorporates a digital app, a paper-based workbook, and one-to-one coaching designed to meet the needs of people with SMI and coexisting type 2 diabetes.

**Conclusions:**

The intervention development work was underpinned by the MoA theoretical framework and incorporated systematic reviews, primary qualitative research, expert stakeholder surveys, and evidence generated during co-design workshops. The intervention will now be tested for feasibility before undergoing a definitive evaluation in a pragmatic randomized controlled trial.

## Introduction

### Health Inequalities

Long-term physical conditions (LTCs), also referred to as chronic diseases, develop earlier and are 2 to 3 times more common in people with severe mental illness (SMI; eg, schizophrenia, schizoaffective disorder, psychosis, and bipolar disorder) than in the general population [1-3]. Life expectancy is substantially reduced by 15 to 20 years among people with SMI [4,5].

The rates of obesity [6], poor diet and nutrition [7], and smoking [8,9] are higher and physical activity levels [10] are lower among people with SMI, likely contributing to the higher prevalence of LTCs and premature mortality in this population. Type 2 diabetes is twice as prevalent in people with SMI, and those with SMI and coexisting type 2 diabetes are more likely to experience poorer clinical outcomes than those with type 2 diabetes alone [11-13]. Reducing the risk of poor clinical outcomes for people with type 2 diabetes is contingent on effective self-management [14-16]. Self-management of type 2 diabetes includes activities such as healthy eating, smoking cessation, stress management, physical activity, blood glucose monitoring, and adherence to medication as prescribed [17]. In England, structured diabetes self-management education programs are recommended for those recently diagnosed with type 2 diabetes, and they provide people with an opportunity to develop their knowledge and skills for self-management [14,16,18]. They are effective in improving self-management behaviors, clinical outcomes, and associated health care costs [19-21].

However, people with SMI and type 2 diabetes rarely receive support for self-management [22]. People with SMI can experience symptoms such as avolition, social withdrawal, or hallucinations, as well as side effects from psychiatric medication, such as fatigue and excessive hunger [23], making it difficult for them to effectively engage in self-management activities. Moreover, it is unclear whether existing diabetes self-management education programs are effective for people with SMI, as they are typically excluded from research on these programs [24]. A Cochrane review of self-management interventions for people with diabetes and SMI included only 1 trial and concluded that there was insufficient evidence about the effectiveness of such interventions in this population [25].

### Digital Exclusion

There is a growing emphasis within health care services on the use of digital technologies, including within services for supporting the self-management of LTCs, such as diabetes [26]. Digital exclusion will likely further exacerbate health inequality among people with SMI [27]. Although digital exclusion for those with SMI is declining in the United Kingdom [28], the proportion of people with SMI who use the internet or computers remains a minority [29], and reasons for nonengagement with digital technology include the lack of access to devices or Wi-Fi, sociodemographic factors, the lack of independence, the lack of skills, and the symptoms of SMI [29]. The COVID-19 pandemic further compounded this inequality, as health services responded to national restrictions by providing services remotely using digital technologies [30]. Those without access to these technologies, including those without the knowledge or skills to use them, and those with symptoms that hinder the use of digital technology faced difficulties accessing health care services during the peak pandemic years [31]. This shift to digital provision of health care services is likely to persist [31], potentially reinforcing preexisting structural and symptom-driven digital exclusion among people with SMI and widening health inequalities among this population.

### Goal of This Study

Therefore, it is essential that any diabetes self-management program for people with SMI addresses the unique needs of people living with both conditions and the inequalities they experience within health care services. We aimed to co-design a self-management intervention for people with SMI and coexisting type 2 diabetes living in the community, incorporating digital technology and paper-based options and addressing the specific barriers that people living with coexisting SMI and type 2 diabetes experience when managing these conditions together.

## Methods

### Overview

We used an approach that integrated behavioral theory and empirical evidence with co-design methods to ensure that the intervention we developed would be effective for improving diabetes self-management in people with SMI, as well as be feasible to deliver and acceptable to both service users and those delivering the intervention. This study was conducted in the United Kingdom, and the approach to co-design was modeled on experience-based co-design, which is primarily a service improvement methodology increasingly used to develop complex interventions; it starts with understanding users’ experiences, needs, and preferences and involves working in partnership with users to design or improve services based on these understandings [32].

**Figure 1:**
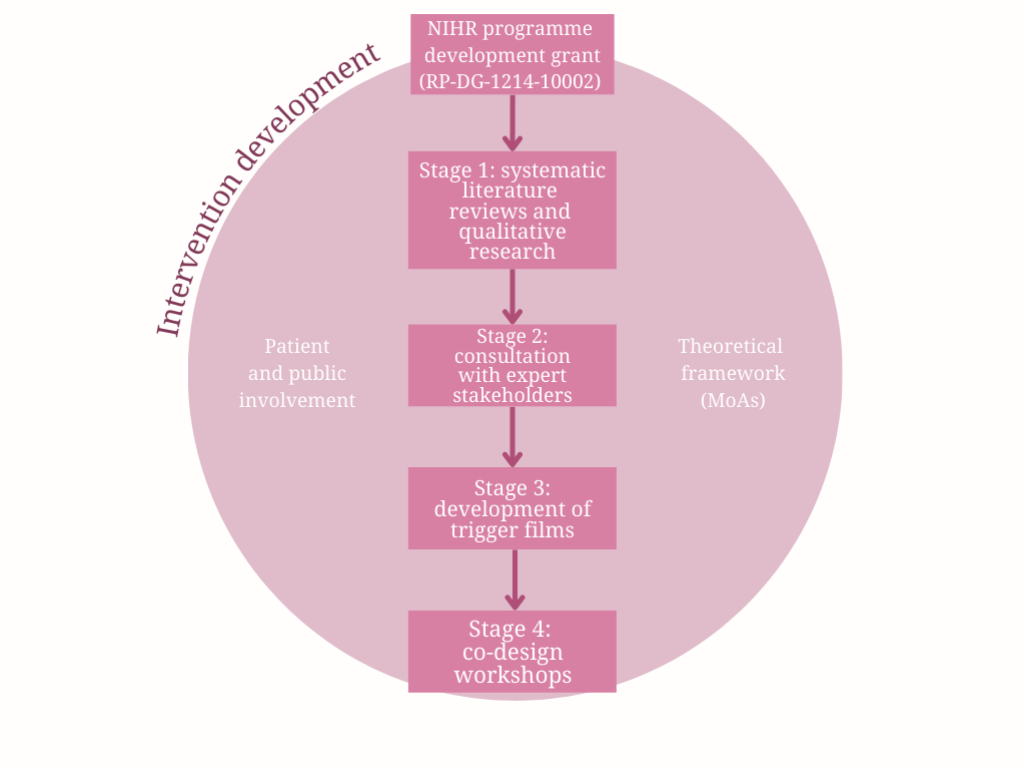
Overview of the intervention development process. MoAs: mechanisms of action; NIHR: National Institute for Health and Care Research.

An overview of the intervention development process is shown in Figure 1. This paper reports the development process for the intervention (stages 2-4), and separate papers report the findings of the systematic reviews [33,34] and qualitative research [23] that formed the basis of stage 1.

### Theoretical Framework

The intervention development process was structured around understanding the mechanisms of action (MoAs) for the self-management behaviors of people with SMI and coexisting LTCs [35]. MoAs are theoretical constructs that are thought to explain the process through which specific behavior change techniques (BCTs) affect self-management [36].

### Patient and Public Involvement

DIAMONDS Voice is a service user and carer group that has contributed to the DIAMONDS (diabetes and mental illness, improving outcomes and self-management) program since 2015. At the time of this co-design study, DIAMONDS Voice consisted of 8 people with SMI and 1 family member. DIAMONDS Voice contributed to the development of the co-design study protocol, attended DIAMONDS research team meetings, and supported the delivery of the study through input on expert consensus surveys, participation in workshops, involvement in synthesis sessions, and design of the intervention materials.

### Program Development Grant

#### Preparatory Research: Underlying Principles and Candidate Intervention Components

The DIAMONDS program is underpinned by a previous work conducted as part of a National Institute for Health and Care Research Programme Development Grant (RP-DG-1214-10002). During this 12-month development period, we conducted a scoping review of the existing diabetes self-management and education programs for people with type 2 diabetes combined with a series of multidisciplinary workshops to explore how to adapt diabetes self-management for people with SMI and conducted 5 patient and public involvement (PPI) panels to build PPI capacity.

The scoping review identified >150 unique interventions for supporting the self-management of diabetes. Although differing in format, duration, and delivery, the interventions were broadly similar in content, and there was no leading candidate approach for adaptation for people with SMI. Many of the interventions from the scoping review were included in a previous systematic review of diabetes self-management interventions by Chrvala et al [37]. This review identified 118 interventions that included goal setting in at least 1 area of self-management and concluded that interventions with a combination of group and individual sessions and those with a mean contact time of >10 hours were more effective than those without these features. We used 2 intervention planning workshops alongside consultation with our PPI panel to obtain feedback on the suitability of different intervention types (eg, group or individual or combined) and to identify how self-management interventions may need to be adapted to meet the needs of people with SMI. The workshops were attended by the DIAMONDS research team and clinical professionals from primary care, diabetes services, and psychiatric services. A total of 19 people attended the first workshop, and 21 people attended the second workshop.

In these workshops, we explored the specific barriers to and facilitators of self-management in people with SMI with experts in the field. Discussions around the educational content of programs concluded that any intervention should cover the 7 main areas of self-management included in the American Association of Diabetes Educators 7 Self-Care Behaviors (healthy coping, healthy eating, being active, taking medication, monitoring, problem-solving, and reducing risks) [17]. The workshops also specified a need for additional one-to-one support, which was not often provided in the existing diabetes self-management and education programs. There were mixed opinions about the extent to which we should use digital technologies and the potential acceptability and feasibility of this approach for people with SMI. The attendees of the workshops also discussed opportunities to train people working in mental health services to deliver a tailored intervention. Diabetes and mental health clinicians, including physicians, specialist nurses, and dietitians, agreed that this approach was feasible. However, they pointed out that although people working in mental health services have the skills to support people with SMI, they may know little about diabetes and would need specific training for this.

PPI panel meetings confirmed that there were mixed opinions regarding the use of digital technologies to support health. The panel agreed that digital applications could form part of an intervention but should not be used to the exclusion of other platforms and intervention content. PPI meetings also identified that in-person support would need to be an essential aspect of any intervention to help reduce anxiety and address low motivation. There was consensus that both group and individual intervention sessions would be needed to support people with SMI and diabetes. There was agreement that group sessions need to be optional and stand-alone and not compulsory so that people could attend if able to.

#### Key Learning Underpinning the Co-design Process

Our preparatory research informed our decision to develop a bespoke self-management intervention for people with type 2 diabetes and SMI. The intervention would need to include both group and one-to-one support to ensure accessibility and engagement for people with SMI. The intervention should also incorporate a digital application, but there should be options available for participants who do not have access to digital technology or who struggle with using it, such as a participant workbook. In addition, the barriers to self-management experienced by people with SMI meant that the intervention would need to be adapted to individual needs. These design principles were taken forward and served as the foundation for a phased approach to intervention development using a co-design methodology.

### Intervention Development Process

#### Stage 1: Primary and Secondary Analysis of Qualitative and Quantitative Evidence About the Determinants of Self-management Behaviors in SMI and LTCs

Stage 1 identified MoAs that were mapped against the determinants of self-management. This stage combined a secondary analysis of published qualitative and quantitative evidence about the enablers of, barriers to, and determinants (mapped against MoAs) of self-management for adults living in the community with SMI and LTCs [33,34] with an analysis of in-depth qualitative interviews with staff, informal caregivers, and service users in England about the lived experience of self-management of LTCs and SMI [23]. Evidential links between all MoAs and the determinants of self-management behavior were cross tabulated. An example of this cross-tabulation mapping the MoA “attitude towards the behaviour” with the determinants identified from the primary qualitative research [23] is shown in Table 1.

**Table 1:** Example of cross-tabulation mapping the mechanism of action attitude toward the behavior to the determinants in primary qualitative research.

Statement	Illustrative quote
I take medication for my mental health by injection which I don’t like because it hurts but it keeps me well	“I have an injection every fortnight and that keeps me stable. I don’t like it, it hurts, it’s horrible but it keeps me well. My husband would tell you, it’s the best thing that’s happened to me” (P^a^).
I get fed up with taking my medications because they cause side effects	“And the Diabetes, I do get fed up with needles every day. Twice a day. In my thigh, twice a day, morning and night. I get fed up with that. And tablets as well, I take a load of tablets every day and I get fed up with that as well. They cause side effects, you see” (P).
I enjoy doing some types of exercise but not others	P: “I used to go the gym.” I^b^: “And how did you find that?” P: “I liked the walking machine and the rowing machine but I didn’t fancy the weights.” I: “Is that something you’d like to carry on doing?” P: “No, not really.” I: “No? Why not?” P: “No, no. I don’t really...I didn’t really enjoy it that much.”
I drink alcoholic drinks because I enjoy it	“Oh I drink about 5 or 6 cans [of alcoholic drinks] a night yeah. To have a good time because I enjoy it, I enjoy it yeah” (P).
I don’t always monitor my blood glucose levels because it seems like too much hard work	“Well, I could do it at tea time I suppose but I don’t because it seems like too much messing about. You’ve got to fiddle about putting this needle thing in here. Put the strip on the machine and then test your blood. Sometimes, when you press the button and no blood comes out, you’ve got to do it again. Stuff like that” (P).
I have a great counsellor that I see every week to help me cope with my conditions	“I go to counselling every week. I’ve got a great counsellor. So, that’s a big part of my self-management” (P).
I used to take part in relaxation sessions which were really good	“I used to do relaxation [at the day center], you know when they put the lights out and listen to music and you relax and they tell you you’re walking through a forest or whatever? That’s really good” (P).
I take part in leisure activities and I like doing them	“There are things I can do, I like to walk as well and there’s some really nice walks around here. Leisure activities, yeah, I can walk the dog and do the garden, I like to paint” (P).

^a^P: participant.

^b^I: interviewer.

#### Stage 2: Consensus and Prioritization Exercise With Stakeholders

Stage 2 involved a 2-phase survey and consultation with expert stakeholders (who participated in stage 1) to prioritize and select candidate BCTs based on established theoretical and empirical evidence about behavior change interventions. Expert stakeholders were first tasked with ranking (on a 4-point Likert scale) the MoAs identified in stage 1 in terms of the perceived strength of their association with the self-management of diabetes and modifiability. A total of 21 people responded to the electronic survey; the stakeholders included health care professionals working in primary care (n=1, 5%), diabetes services (n=5, 24%), chronic obstructive pulmonary disease care (n=4, 19%), and mental health services (n=2, 10%); clinical academics (n=3, 14%); carers (n=1, 5%); service users (n=4, 19%); and health care managers (n=1, 5%).

The DIAMONDS research team then mapped the average expert-rated rankings for MoA-BCT associations with the cross-tabulation mapping from stage 1 to generate a list of candidate MoAs and BCT links. The second phase of the survey was administered again to the expert stakeholders approximately 2 months after the completion of the first survey to judge each MoA and BCT link according to whether it was acceptable and practical using the APEASE (Affordability, Practicability, Effectiveness, Acceptability, Safety and Equity) criteria. The BCTs and MoAs that the stakeholders reached a consensus on were carried forward to inform the potential content in the co-design process.

For each MoA, between 4 and 11 BCT links were identified. Following the prioritization process, the DIAMONDS research team selected the BCTs that offered the most promise in terms of potential efficacy, acceptability, and deliverability using the results of both surveys, preparatory work, expert consultation, and PPI. The 22 BCTs that were included in the intervention are listed in Table 2.

**Table 2:** Included behavior change techniques (BCTs) and linked mechanisms of action (MoAs).

BCTs	Linked MoAs
Goal setting (behavior)	Intentions; goals; and memory, attention, and decision processes
Problem-solving	Knowledge; skills; beliefs about capabilities; memory, attention, and decision processes; and behavioral regulation
Action planning	Memory, attention, and decision processes; behavioral regulation; and behavioral cueing
Graded tasks	Skills; beliefs about capabilities; and memory, attention, and decision processes
Review behavior goals	Beliefs about capabilities, reinforcement, intentions, goals, and feedback processes
Feedback on behavior	Reinforcement, motivation, and feedback processes
Focus on past success	Beliefs about capabilities
Prompts or cues	Memory, attention, and decision processes; environmental context and resources; behavioral regulation; and behavioral cueing
Habit formation	Memory, attention, and decision processes; behavioral regulation; and behavioral cueing
Information about health consequences	Knowledge, beliefs about consequences, intentions, and attitude toward behavior
Information on how to perform the behavior	Knowledge, skills, and beliefs about capabilities
Demonstration of the behavior	Knowledge, skills, and beliefs about capabilities
Body changes	Beliefs about capabilities
Verbal persuasion about capability	Beliefs about capabilities
Self-monitoring of behavior	Behavioral regulation, feedback processes, and behavioral cueing
Feedback on behavior	Reinforcement, motivation, and feedback processes
Reduce negative emotions	Skills, emotion, and behavioral regulation
Self-monitoring the outcome of behavior	Goals
Monitoring of emotional consequences	Beliefs about consequences and emotion
Information about emotional consequences	Knowledge, beliefs about consequences, and emotion
Social support (unspecified)	Environmental context and resources
Social support (practical)	Environmental context and resources

#### Stage 3: Development of Trigger Films

Stage 3 happened in parallel with the first 2 stages and involved the creation of animated trigger films. Trigger films, also known as catalyst films, are films designed to generate discussion on a specific topic [32]. The films were based on key themes and illustrative points from our qualitative study [23] and qualitative evidence synthesis [34] about the experiences of people with coexisting SMI and LTCs, their informal caregivers, and health care professionals. These themes included the impact of SMI on self-management behaviors, interaction between SMI and LTCs, and barriers to and facilitators of self-management. During stage 3, we worked with an external collaborator, the digital design agency HMA (HMA Digital Marketing Ltd) [38]. We used animations within the trigger films to integrate and illustrate various themes and points rapidly. Subsequently, we used these trigger films and the personas we developed in stage 4 as part of the co-design process. We developed 2 trigger films, one consisting of an overview of 3 characters who had SMI and LTCs and another providing different self-management scenarios for each of the 3 characters to highlight the difficulties people with SMI experience when trying to manage their health.

#### Stage 4: Co-design Workshops With Staff, Service Users, and Carers

Stage 4 included an initial discovery day workshop that lasted 5.5 hours, followed by 5 co-design workshops with staff and service users over 8 months. The 5 co-design workshops took place face-to-face and lasted approximately 15 hours in total. The co-design workshops were facilitated by mHabitat (currently Thrive by Design Ltd [39]) and were attended by our digital design partner HMA [37]. During the workshops, the DIAMONDS research team, digital design partner, service users, and health care professionals worked closely together to inform the development of the intervention.

#### Co-design Participants

Workshop participants were recruited from three groups: (1) service users (ie, individuals with SMI and LTCs); (2) informal caregivers; and (3) health care professionals involved in the care of people with SMI, LTCs, or both.

We recruited participants from the existing DIAMONDS research cohorts (ie, people who had taken part in the program development work or related research and had given consent to be contacted again), DIAMONDS Voice, and 3 National Health Service (NHS) mental health trusts in Yorkshire, United Kingdom. The eligibility criteria for workshop participants are listed in Textbox 1.

All potential participants received an invitation pack about the study via post, including an invitation letter, a participant information sheet, and an expression of interest form that included consent to contact. The DIAMONDS research team contacted individuals who expressed an interest and explained the study to them. All participants signed a consent form at the beginning of the first discovery day for the duration of the co-design workshops. Overall, 24 people were recruited for stage 4 of the study. Of them, 8 (33%) people were recruited and provided consent but were unable to attend any of the workshops, 7 (88%) of whom were health care professionals, and 1 (12%) was a service user. One of the health care professionals attended the first discovery day workshop and then withdrew from the study. The number of attendees by group for each workshop is listed in Table 3.

**Eligibility criteria for workshop participants.**

- Service users
  People who have coexisting severe mental illness (SMI) and type 2 diabetes were eligible. People were excluded if they were in an acute psychiatric ward during the study recruitment period, if they lacked the capacity to participate, or if they had experienced a recent relapse of their SMI. People with a diagnosis of gestational diabetes only were also excluded.
- Informal caregivers
  People who provided informal support to people with coexisting SMI and type 2 diabetes, including family members and friends, were eligible.
- Health care professionals
  People who had professional experience supporting people with either SMI or type 2 diabetes were eligible.

**Table 3:** Overview of the workshop participants (N=16).

Workshop and participant designation		Participant, n (%)	
**Discovery day (n=9)**			
	Service users	3 (33)	
	Informal caregivers	2 (22)	
	Health care professionals	4 (44)	
**Workshop 1 (n=6)**			
	Service users	3 (50)	
	Informal caregivers^a^	3 (50)	
	Health care professionals	0 (0)	
**Workshop 2 (n=8)**			
	Service users^a^	4 (50)	
	Informal caregivers	2 (25)	
	Health care professionals	2 (25)	
**Workshop 3 (n=10)**			
	Service users^a^	5 (50)	
	Informal caregivers^a^	3 (30)	
	Health care professionals	2 (20)	
**Workshop 4 (n=9)**			
	Service users^a^	4 (44)	
	Informal caregivers^a^	3 (33)	
	Health care professionals	2 (22)	
**Workshop 5 (n=5)**			
	Service users	3 (60)	
	Informal caregivers	2 (40)	
	Health care professionals	0 (0)	

^a^Including a member of DIAMONDS Voice.

#### Discovery Day Workshop

The discovery day workshop was split between separate morning sessions for staff and service users and a joint afternoon session. To address the barriers to using a digital application to support self-management, the participants were asked to write down their frustrations surrounding digital technology on Post-it notes and place these on a “wall of frustration,” which included a poster illustrating participant quotes about experiences with digital technology from our qualitative research [23]. Following this initial exercise, the trigger films were shown separately to each group.

The service user group was asked to complete a journey mapping activity, mapping how a typical day in their life compared with and related to the characters in the trigger films. Following journey mapping, the service users were invited to identify problematic moments they experienced during a typical day when trying to manage their health. During this activity, the service user participants expressed that their ability to engage in self-management behaviors was heavily compromised by their mental health symptoms. Furthermore, as people with SMI, the service users described how they often felt intimidated by going to public places, such as gyms, and that this acted as a barrier to physical activity. The service users also identified that deciding where to do food shopping and their ability to cook were important considerations that underpinned their ability to manage their physical health.

**Figure 2:**
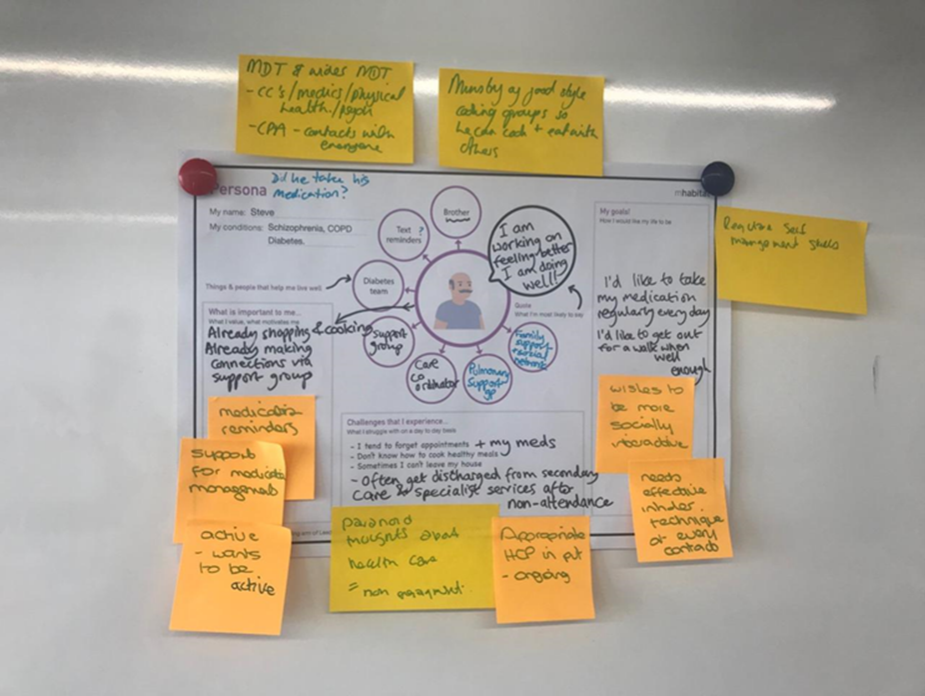
Outputs from the discovery day workshop persona activity.

The staff group was asked to draw up a care pathway for service users with SMI and type 2 diabetes and to determine whether there were any points along this pathway where digital technology could be incorporated. Following these separate discovery sessions, service users and health care professionals were brought together for a joint discovery exercise. The health care professional group presented the pathway they had designed, whereas the service user group presented an outline of “a day in the life of” to highlight the self-management activities they engaged in each day. The service users highlighted the challenges they often experienced, such as access to health care services and the acceptability of the services provided. Health care professionals recommended that optimized care pathways for people with SMI should consider the inclusion of structured education about type 2 diabetes. Figure 2 captures the perspectives of the workshop participants about the challenges of living with SMI and LTCs.

In the joint final session on the discovery day, the 2 groups reviewed the outputs from the earlier sessions. Variable sleep patterns, taking medication, and eating were highlighted as issues that contributed to a person’s ability to engage in self-management. Mental health was identified as having a much greater impact on quality of life and health and, therefore, was typically prioritized over the self-management of type 2 diabetes.

#### Discovery Day Synthesis

Following the discovery day, an in-person synthesis session was held to develop paper prototype components for a self-management intervention that could be delivered within an app or a workbook with prioritized functions and placeholder content. This prototype was based on a synthesis of the information gained during the discovery day and the MoAs and BCTs identified in stages 1 and 2. The prototype was used as a framework for the following co-design workshops.

During the synthesis session, the research team, DIAMONDS Voice, and staff from HMA and mHabitat categorized the notes from the discovery day as follows: (1) content (eg, smoking cessation), (2) functionality (eg, any interaction with the intervention), (3) insights (into the behavior of people with SMI), and (4) risk (eg, clinical or data risk).

Next, the group collaboratively prioritized content in the following order:

1. Motivation/daily routine (including sleep)
2. Healthy eating
3. Physical activity/exercise
4. Stress management/emotion/mood
5. Taking medication

For each of the prioritized functions, the workshop participants explored how that function could be delivered. For example, the development of a daily routine could be supported by self-monitoring, consisting of prompts within an app.

#### Co-design Workshops

A total of 5 co-design workshops were conducted after the discovery day and discovery day synthesis session. At least 1 member of the DIAMONDS research team attended each workshop to observe, take notes, and provide information about the existing evidence. Workshop content was developed iteratively based on a priori objectives, preceding synthesis sessions, and both verbal (in-person) and written inputs from the wider DIAMONDS team. Photographs of the workshop outputs were used as a basis for the synthesis sessions, during which the DIAMONDS team and DIAMONDS Voice iteratively tested and refined intervention prototypes, which included both the digital app and the manualized paper-based content.

##### Workshop 1: Testing of a Wireframe of the Prototype and Prioritization of the Target Health Behaviors

Bringing together discussions and learning from the previous steps, a paper prototype of an app entitled “Change One Thing” was presented to the participants. The prototype focused on supporting small lifestyle changes, hence the name “Change One Thing.” It was presented using a series of questions (eg, Do you sleep well?) and designed to offer daily prompts for a chosen activity (eg, for going to sleep at a regular time).

The participants were divided into 2 groups: a mix of service users and informal caregivers. One group was asked to provide feedback on the prototype, whereas the other group was asked to rank by priority 5 self-management behaviors that had been identified from stages 1 to 4, with number 1 being the highest priority (ie, the most important) and number 5 being the lowest priority (ie, the least important). Medication and managing sleep were the top priority self-management behaviors for most of the participants.

The participants felt that the app would help them remember to take their medication, and the inclusion of videos and the ability to track progress could motivate them to engage in self-management. The participants also discussed the use of one-to-one and group sessions to deliver the intervention. They agreed that one-to-one sessions would be necessary for engagement and help people work toward specific goals related to healthy eating, exercise, and sleep, whereas group sessions were considered to add social support.

##### Workshop 2: Ideation and Prototype Sense Checking

The participants were shown ideas for the “look and feel” of the intervention materials (app and workbook), the initial setup process for the app, and the process for goal setting and action planning. Discussion about the appearance, setup, and utility of the app was facilitated in relation to exemplar content about stress management and physical activity.

Participants reviewed 5 potential options for the look and feel of the “Change One Thing” app and workbook and identified preferred questions for supporting goal setting and action planning. Using the key priorities identified in workshop 1, suggested goals were provided for medication and sleep, with options for how these goals could be phrased and monitored. Additional content for the workbook and app was suggested for how people could add personalized changes to physical activity (eg, walking to the postbox) and how people could seek social support to manage stress. The participants placed an emphasis on person-centered content that considered personal ability and interests, with an intervention facilitator asking questions rather than making suggestions.

##### Workshop 3: Refined Prototype Sense Checking

The proposed components included 16 weekly one-to-one meetings, a digital app or a workbook entitled “Change One Thing,” and monthly group sessions. These components were informed by the preparatory research that was conducted during the program development grant, alongside the information and preferences provided during the discovery day and first 2 workshops and input from DIAMONDS Voice members.

The workshop participants were split into 2 groups: one group of health care professionals and one group of service users and informal caregivers. The health care professional group focused on the training and support needs of intervention facilitators. The proposed support included supervision, training on diabetes and mental health, goal setting, and personalization of care. Health care professionals also identified the need for processes to be set up around referrals and reporting for participants who may be at risk or who may become unwell while taking part in the intervention.

The service user group reviewed the specificity and emotional valence of the language and wording proposed for the initial goal-setting and action planning activities, which would be completed either in the “Change One Thing” app or workbook. The participants suggested alternative goal statements, which were not appropriate, as they could not be framed within action plans or contradicted the evidence base (eg, sleeping in on the weekend instead of having a regular sleep schedule). The service user and health care professional groups then reconvened and provided feedback on their activities.

##### Workshop 4: User Acceptance Testing and Evaluation

**Figure 3:**
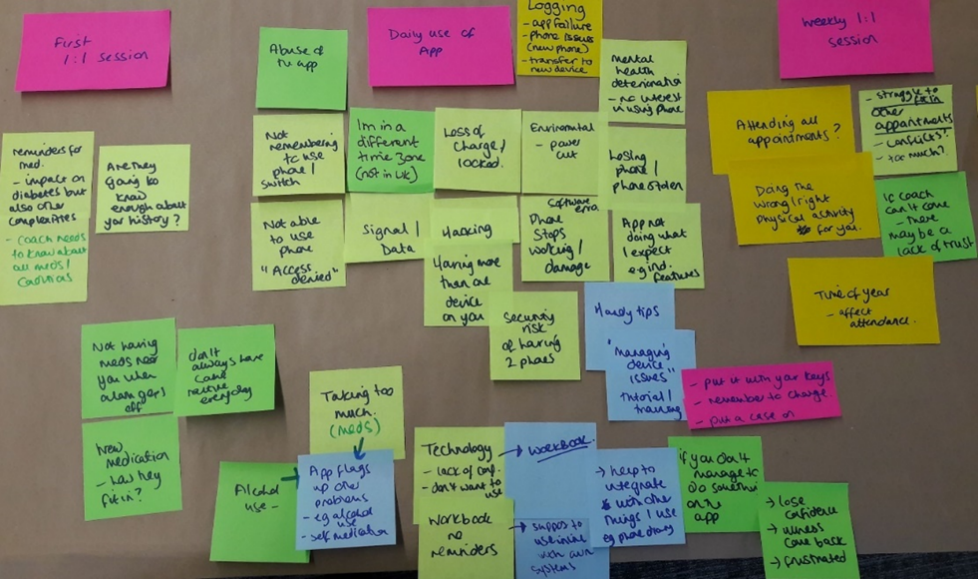
Solution-focused evaluation of the intervention components.

This workshop focused on the clinical safety of the intervention. Topics of concern addressed the problems associated with using the “Change One Thing” app and workbook, the one-to-one sessions, and the end of the intervention. The problems associated with using the app included concerns about the security of personal data, malfunctioning of the app, and phone charging. These could be best solved by ensuring that the workbook delivers the same content and function as the app. The participants also identified that after the final intervention sessions, people will need to be signposted to relevant support services. A photograph of this evaluative process, outlining the concerns and potential solutions associated with the first sessions, daily use of the app, and weekly one-to-one sessions, is shown in Figure 3.

##### Workshop 5: Further User Acceptance Testing and Evaluation to Inform the Refinement of the Intervention

User testing and evaluation in workshop 4 were truncated because of a fire alarm; therefore, we repurposed workshop 5 to complete these processes. The participants watched a series of educational animations that had been specifically created for inclusion in the “Change One Thing” app and workbook (presented in the workbook as comic strips) and were asked to provide feedback about the acceptability, utility, and relevance of each video. The videos focused on sleep, medication taking, fluid intake, and physical activity and were based on the personas previously shown in the trigger films during the discovery day. After viewing the videos, the participants were asked a series of questions to review their understanding of the educational content. The participants demonstrated a good understanding of what the videos were trying to communicate and provided suggestions on how they could be improved.

Digital app user testing sessions were facilitated by the research team and ran in parallel with the review of the educational animations. The participants were asked to use the app and provide feedback on its functionality and content. Despite being smartphone users, several participants found it difficult to use the app. The problems that were encountered included repeated crashing or locking of the screen and difficulties using some of the features, such as setting the time or using sliders within the app. Therefore, it was recommended that service users be supported by the intervention facilitators to set up the app and enable accessibility options during the onboarding process.

#### Design of the Intervention Materials

The main outputs from the co-design process were the Change One Thing app and workbook and the DIAMONDS coach manual and training handbook. We hosted several collaborative meetings with Nifty Fox Creative (Nifty Fox Creative Ltd) [40] and DIAMONDS Voice to ensure that the layout, language, design, and organization of the workbook were accessible, engaging, fit for purpose, and matched the content of the app. We worked with the Leicester Diabetes Centre [41] to design the coach manual and training handbook. The Change One Thing app was designed and developed during the co-design process by our digital design partner HMA [38] and finalized by the app developer Pipe and Piper (Pipe & Piper Ltd) [42].

### Ethics Approval

Ethics approval was obtained from the North West–Greater Manchester West Research Ethics Committee (reference: 19/NW/0356).

## Results

### Intervention Overview and Structure

The DIAMONDS intervention aims to support the self-management of type 2 diabetes for people with coexisting SMI by improving diabetes self-management knowledge and skills and enhancing the specific capabilities, opportunities, and motivations that influence diabetes self-management in this population. In particular, the intervention provides tailored and person-centered support for setting behavioral goals, action planning, problem-solving, and increasing physical activity and provides and facilitates peer support. The logic model for the DIAMONDS intervention is provided in Multimedia Appendix 1.

**Figure 4:**
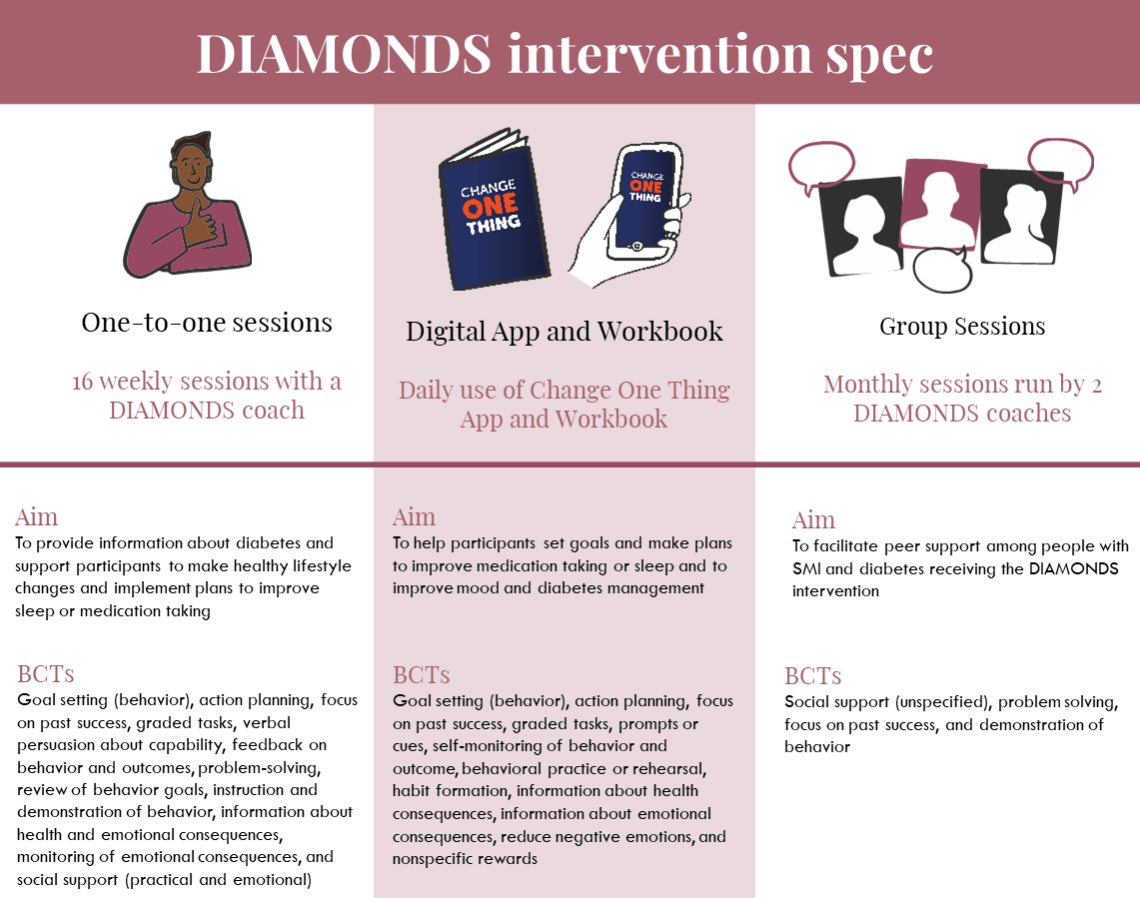
The DIAMONDS (diabetes and mental illness, improving outcomes and self-management) intervention. BCT: behavior change technique; SMI: severe mental illness.

The DIAMONDS intervention will be delivered by a trained facilitator called a “DIAMONDS Coach.” The participants will be offered weekly sessions either face-to-face, over the phone, or over video call with their DIAMONDS Coach for up to 16 weeks, in combination with the daily use of the DIAMONDS workbook or the DIAMONDS app called “Change One Thing.” They can also engage in optional in-person monthly group sessions facilitated by 2 DIAMONDS Coaches, with other participants receiving the intervention. We will establish the feasibility and acceptability of the intervention before evaluating its clinical effectiveness and cost-effectiveness in a randomized controlled trial [43]. An overview of the core intervention components is presented in Figure 4.

### Delivering the BCTs: Goal Setting

As outlined in the logic model provided in Multimedia Appendix 1, the intervention aims to deliver several BCTs to support people in making lifestyle changes that will support their physical health. To demonstrate how these BCTs will be delivered using the Change One Thing app or the workbook, we will outline the BCT goal setting within the DIAMONDS intervention. In each one-to-one session, the DIAMONDS Coach will follow the subsequent steps to support the participant in setting a goal.

**Figure 5:**
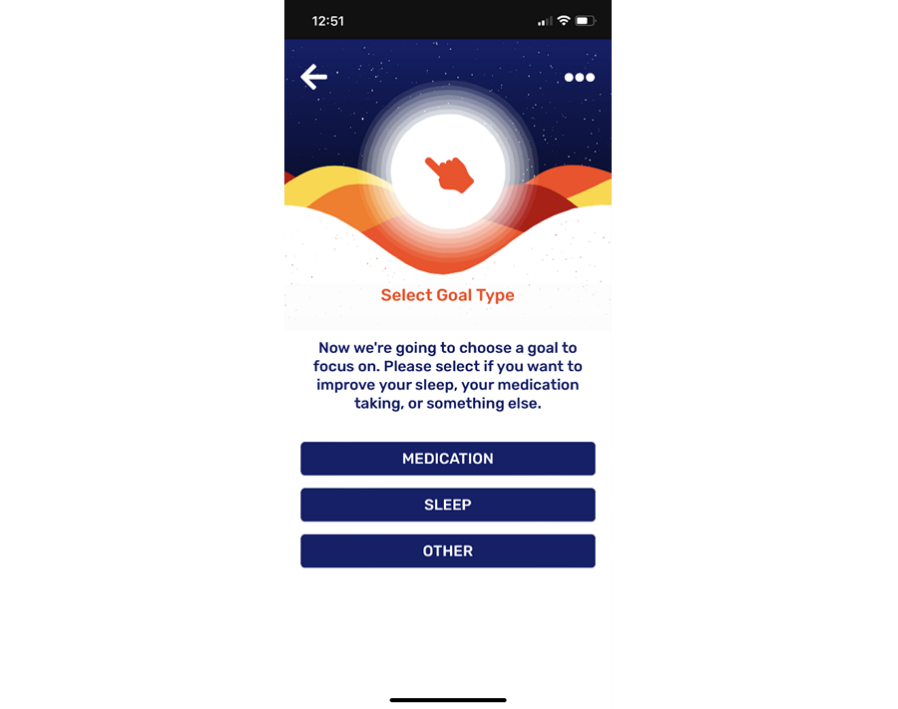
Goal types within the Change One Thing app.

**Figure 6:**
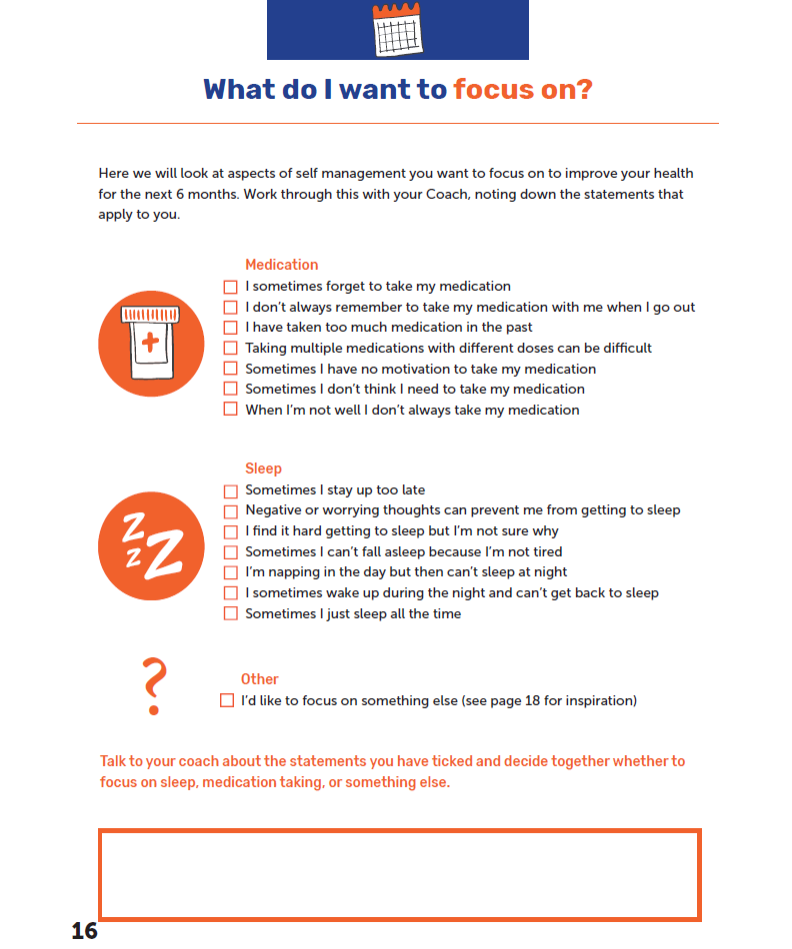
Goal types within the workbook.

Using the app or workbook, the DIAMONDS Coach will ask the participants what they would like to focus on during the following week. The app and workbook have been designed to focus on goals relating to sleep and taking medication, as these were identified during the development process as the main priorities for service users. However, the participants may want to focus on a different problem and, therefore, can identify their own goal if preferred. Examples of these choices in the app and workbook are shown in Figures 5 and 6, respectively.

Once the participant has identified what they want to focus on, the DIAMONDS Coach will support them in developing a goal. If the participant is using the Change One Thing app and chooses to focus on medication or sleep, the goal statement will be automatically generated. If the participant is using the participant workbook, they can choose from a list of goal statements, which are identical to those generated in the app, related to the chosen behavior—medication or sleep. If the participant has not identified medication or sleep as the behavior that they want to focus on, the DIAMONDS Coach will support them in generating a goal statement related to the other behavior they have identified (eg, healthy eating), ensuring that the goal is realistic and achievable.

The goal will be automatically recorded in the app once it has been generated by the participant. If the participant uses the workbook, then the participant or DIAMONDS Coach will record the goal in the goal-setting section of the workbook.

## Discussion

### Principal Findings

This paper describes how we integrated an evidence-based intervention development approach and a co-design methodology to develop a complex intervention for supporting people with SMI and type 2 diabetes in self-managing their health. Drawing on the philosophy that intervention development is best served by integrating theory, evidence, and person-centered approaches [44], we set out to combine behavioral theory with empirical evidence about the determinants of self-management behaviors and user-centered design. A consistent thread in our development work has been the use of the MoA framework to underpin our research on self-management behaviors in people with SMI and LTCs, combined with the use of the BCT taxonomy as a means of linking determinants to intervention components. This theoretical lens has offered a means to interpret systematic review evidence about the determinants of self-management behaviors in people with SMI and diabetes [33]. Our understanding of the drivers and lived experiences of managing LTCs in the context of SMI was further extended by primary qualitative exploration and qualitative evidence synthesis [34]. In this sense, we were able to ensure that the first phases of the co-design process were founded on a firm theoretical and evidential basis, leading to more targeted and person-centered co-design workshops.

Person-centered approaches revolved around thinking about how end users would interact with and use the intervention. End users of the DIAMONDS intervention are not only service users with SMI and diabetes (and their informal caregivers) but also health professionals tasked with intervention delivery. The consistent involvement of end users throughout the co-design process was critical to managing tensions between developing an intervention that could be feasibly delivered for a clinical trial (with the primary goal of reducing glycated hemoglobin [HbA_1c_]) and developing an intervention that met the priorities of service users. For example, service users highlighted their desire to focus on mental health, whereas health care professionals felt that it was important to incorporate physical activity as a means of reducing HbA_1c_. Previous research has highlighted that the management of mental health is frequently the priority for people with SMI over the self-management of LTCs [23]. Trials of other bespoke self-management interventions for people with SMI have failed to demonstrate an effect on clinical biomarkers, despite improving other measures that may be of a higher priority to people with SMI, such as hospitalization [45]. By co-designing an intervention that accommodates goals for both physical and mental health, for example, by framing physical activity as a method of improving mood, we have potentially offered a solution to overcome the disconnect between the priorities of service users and clinical imperative to reduce HbA_1c_.

In addition, our person-centered approach was key to the decision-making about including a digital component in the final intervention specification. People with SMI are less likely to have access to digital technology [28] and face more barriers to its use when they do have physical access (eg, lacking necessary skills) [46], but there is evidence that digital health technologies can feasibly support people with SMI [47] and that access to the internet and smartphones is increasing in this population, particularly in younger age groups [48]. To minimize digital exclusion, we, therefore, developed the “Change One Thing” app but built in user flexibility by ensuring that the same components are provided in the workbook.

Although the effectiveness of this self-management intervention has yet to be demonstrated, our approach to co-designing embraced innovation processes likely to be critical to its future scalability. By involving different stakeholders from different professional roles in the design process, we recognized that digital interventions especially need to be co-created in the context of existing and future service delivery models and work practices [49]. In the English NHS, the establishment of integrated care systems offers opportunities to scale interventions such as the DIAMONDS intervention beyond the local level and reach the needs of populations [50]. A key enabler here will be identifying a workforce able to support the onboarding and use of the digital content of this intervention. As part of the new job roles linked to the delivery of the NHS Long Term Plan, it is possible that health and well-being coaches could be trained to support the access to and use of the DIAMONDS intervention, including the Change One Thing app. Social support that can build trust about the safety and utility of the intervention among users is also a known enabler of scaling up digital interventions [51]. Going forward, we will conduct a process evaluation within the definitive trial, exploring the barriers to and facilitators of the implementation of the DIAMONDS intervention. This process evaluation will consider the role of informal caregivers in the delivery of the intervention and whether they are crucial resources for wider scalability beyond the trial context. In these ways, our co-design study is embedded within a broader program of work that is necessarily anticipating how to address the barriers to the future scalability of the intervention.

### Conclusions

We adopted an inclusive and a participatory approach to co-designing a flexible and user-focused intervention for support the self-management of physical and mental health in people with SMI and diabetes. We integrated behavioral science theory, empirical evidence, and co-design approach to structure the intervention development process, informed by our linked work to model and evidence the barriers to and facilitators of self-management behaviors in this population. This approach shows the value of combining behavioral theory with critical insights from primary and secondary research to maximize the utility and success of co-design with diverse groups of stakeholders.

## Appendix

Multimedia Appendix 1 Logic model.

## Abbreviations

APEASE: Affordability, Practicability, Effectiveness, Acceptability, Safety and Equity
BCT: behavior change technique
DIAMONDS: diabetes and mental illness, improving outcomes and self-management
HbA_1c_: glycated hemoglobin
LTC: long-term condition
MoA: mechanism of action
NHS: National Health Service
PPI: patient and public involvement
SMI: severe mental illness
