# Supplementary material for: Development of a Supported Self-management Intervention for People With Severe Mental Illness and Type 2 Diabetes: Theory and Evidence-Based Co-design Approach
Source: J Med Internet Res. 2023 May 12;25:e43597. doi: 10.2196/43597 (PMC10221514; doi:10.2196/43597)
Supplement: Multimedia Appendix 1 [file jmir_v25i1e43597_app1.docx]

| Resources | *Activities*  BCTs from BCTTv1 | Outputs | Outcomes | | | Impact |
| --- | --- | --- | --- | --- | --- | --- |
|  |  |  | Short- term (MoAs) | Medium-term (Behaviours) | Long-term (Health changes) |  |
| Session 1 (onboarding) – facilitated by Change One Thing *app* and *workbook* | ***Information Giving***  4.2 Information about antecedents  ***Select goal and action plan***  1.1 Goal setting behaviour  1.4 Action plan  7.1 Prompts & cues  ***Physical activity planning***  5.6 Information about emotional consequences  15.3 Focus on past success  ***Planning for good days and bad days***  1.2 Problem solving  11.2 Reducing negative emotion  ***Specify contacts***  3.1 Social support unspecified  3.2 Social support practical  3.3 Social support emotional | Evidence of fidelity of delivery by Coach (intervention fidelity checklist)    App downloaded by participant  Workbook set up | Behavioural regulation  Goals  Behavioural cueing  Memory, attention, and decision processes  Social influence  Beliefs about consequences  Beliefs about capabilities | Engagement with/adherence to a personalised self-management action plan with the aim to make sustainable lifestyle changes (e.g., sleep, medication taking, physical activity) | Primary outcome: Glycaemic control (HbA1c)  Secondary outcomes:  Physical activity  Psychological health  Cholesterol  Haemoglobin  BMI  Blood pressure  Diabetes complications  Health resource use  Smoking status | Mental health  Wellbeing  Diabetic complications  Healthcare costs/use  QoL  Service delivery |
| Maintenance sessions – facilitated by Change One Thing *app* and *workbook* | ***Review progress of previous week***  1.2 Problem solving  1.5 Review behavioural goals  2.2 Feedback on behaviour  2.7 Feedback on outcomes of behaviour  15.3 Focus on past success  ***Goal setting and action planning***  1.1 Goal setting behaviour  1.4 Action plan  7.1 Prompts & cues  8.7 Graded tasks  ***Physical activity planning***  5.6 Information about emotional consequences  8.7 Graded tasks  15.3 Focus on past success  ***Planning for good days and bad days***  1.2 Problem solving  11.2 Reducing negative emotion  ***Diabetes education***  4.1 Instructions on how to perform a behaviour  4.2 Information about antecedents  5.1 Information about health consequences  5.6 Information about emotional consequences  8.3 Habit formation  9.1 Credible source | Evidence of fidelity of delivery by Coach (intervention fidelity checklist)  Workbook completion  App use | Behavioural regulation  Feedback processes  Beliefs about capabilities  Goals  Behavioural cueing  Memory, attention, and decision processes  Beliefs about consequences |  |  |  |
| Step-down session – facilitated by Change One Thing *app* and *workbook* | ***Review progress of previous week(s)***  1.2 Problem solving  1.5 Review behavioural goals  2.2 Feedback on behaviour  2.7 Feedback on outcomes of behaviour  15.3 Focus on past success  ***Review understanding of diabetes***  4.2 Information about antecedents  5.6 Information about emotional consequences  5.1 Information about health consequences  4.1 Instructions on how to perform a behaviour  9.1 Credible source  8.3 Habit formation  ***Identify existing support structures***  3.1 Social support unspecified  3.2 Social support practical  3.3 Social support emotional | Evidence of fidelity of delivery by Coach (IF checklist)    App use  Workbook completion | Motivation  Feedback processes  Knowledge  Behavioural regulation  Beliefs about consequences |  |  |  |
| Workbook (used between sessions) | ***Progress tracking***  2.3 Self-monitoring of behaviour  2.4 Self-monitoring of outcomes of behaviour | Workbook completion | Behavioural regulation |  |  |  |
| App  (used between sessions) | ***Progress tracking***  2.3 Self-monitoring of behaviour  2.4 Self-monitoring of outcomes of behaviour | App use | Behavioural regulation |  |  |  |
